# Diabetes severity is strongly associated with the risk of active tuberculosis in people with type 2 diabetes: a nationwide cohort study with a 6-year follow-up

**DOI:** 10.1186/s12931-023-02414-5

**Published:** 2023-04-11

**Authors:** Ji Young Kang, Kyungdo Han, Seung-Hwan Lee, Mee Kyoung Kim

**Affiliations:** 1 Division of Pulmonology, Department of Internal Medicine, Cheju Halla General Hospital, Jeju, 63127 Korea; 2 Department of Statistics and Actuarial Science, Soongsil University, Seoul, 06978 Korea; 3 Division of Endocrinology and Metabolism, Department of Internal Medicine, Seoul St. Mary’s Hospital, College of Medicine, The Catholic University of Korea, #222 Banpo-daero, Seocho-Gu, Seoul, 06591 Korea; 4 Division of Endocrinology and Metabolism, Department of Internal Medicine, Yeouido St. Mary’s Hospital, College of Medicine, The Catholic University of Korea, #10 63-Ro, Yeongdeungpo-Gu, Seoul, 07345 Korea

## Abstract

**Background:**

Many have the rising coincidence of diabetes mellitus (DM) and endemic tuberculosis (TB). We evaluated whether the severity of diabetes is associated with an increased risk of active TB infection.

**Methods:**

Using a nationally representative database from the Korean National Health Insurance System, 2, 489, 718 people with type 2 DM who underwent a regular health checkup during 2009–2012 were followed up until the end of 2018. The diabetes severity score parameters included the number of oral hypoglycemic agents (≥ 3), insulin use, diabetes duration (≥ 5 years), and the presence of chronic kidney disease (CKD) or cardiovascular disease. Each of these characteristics was scored as one point, and their sum (0–5) was used as the diabetes severity score.

**Results:**

We identified 21, 231 cases of active TB during a median follow-up of 6.8 years. Each parameter of the diabetes severity score was associated with an increased risk of active TB (all P < 0.001). Insulin use was the most significant factor related to the risk of TB, followed by CKD. The risk of TB increased progressively with increasing diabetes severity score. After adjusting for possible confounding factors, the hazard ratio (95% confidence interval) for TB were 1.23 (1.19–1.27) in participants with one parameter, 1.39 (1.33–1.44) in those with two parameters, 1.65 (1.56–1.73) in those with three parameters, 2.05 (1.88–2.23) in those with four parameters, and 2.62 (2.10–3.27) in those with five parameters compared with participants with no parameters.

**Conclusion:**

Diabetes severity was strongly associated in a dose-dependent manner with the occurrence of active TB. People with a higher diabetes severity score may be a targeted group for active TB screening.

**Supplementary Information:**

The online version contains supplementary material available at 10.1186/s12931-023-02414-5.

## Introduction

Along with the major risk factors for tuberculosis (TB), such as current smoking, heavy alcohol consumption, and malnutrition, diabetes mellitus (DM) is a main contributor to the risk of TB [1–3]. The recent increase in the incidence of DM at the same time as a slow decline in TB incidence has led to the co-occurrence of DM and TB in an estimated 1 million people worldwide [2]. The percentage of new cases of TB with co-occurrence of DM is expected to increase substantially from 11.4% in 1990 and 21.9% in 2017 to 33.3% in 2050 [3]. By 2050, at least one-third of TB incidence and almost half of TB mortality in Asia–Pacific countries will be attributed to DM [3]. In addition, the COVID-19 pandemic has had adverse effects on TB and DM management [4, 5]. However, it seems difficult to apply preventive policies fully considering the large population of people with DM worldwide and that most people with TB and DM live in insufficient economic countries. It is important to identify subjects requiring targeted screening or therapies for active TB among diabetic patients and to provide a major benchmark for efficient healthcare services.

The clinical manifestations of more severe diabetes are a consequence of diverse and complex pathophysiological processes affecting different organ systems over time, making it difficult for a single severity measure to capture this complexity adequately [6]. Prior studies on diabetes severity used a grading system based on diabetes-related complications and glycemic control measures [6]. In current clinical practice, glycated hemoglobin (HbA1c) level over the preceding 3 months is used as an indicator of glycemic control and may provide a simple proxy measure of disease severity and to guide interventions and management. However, the ability to estimate the severity of DM using HbA1c level recorded at a specific time point is limited because this level tends to fluctuate over time [6]. In the general treatment flow, people with type 2 DM who have difficulty managing glycemic control with one or two oral hypoglycemic agents (OHAs) may require three or more OHAs or insulin initiation. Therefore, the severity of DM is also evaluated in terms of the number of OHAs [7] or insulin treatment [8]. We have devised a diabetes severity score to provide a comprehensive evaluation of DM status. Here, we explored the association between diabetes severity and the development of active TB in nationwide cohort data with a ≥ 6-year follow-up.

## Methods

### Participants

We used the Korean National Health Insurance Service (NHIS) datasets of claims and health checkups from January 2009 to December 2012. The Korean NHIS is a single-payer insurance organization managed by the Korean government and covers all residents in Korea. The NHIS claims database includes a deidentified research dataset of demographic information, primary and secondary diagnoses classified according to the International Classification of Diseases, 10th Revision (ICD-10), prescriptions, procedures, hospital arrival route, date of admission, and duration of hospitalization for all residents of Korea [9, 10].

All examinees were requested to have biannual health checkups. The results from these health examinations are compiled into preventive health checkup datasets, which constitutes the largest nationwide cohort database of laboratory information in Korea.

In this study, people aged ≥ 20 years who underwent a national health checkup between January 2009 and December 2012 (index year) were selected. Among those who underwent a health examination in 2009–2012 (index year), 2,745,638 who had type 2 DM were included in this study. Type 2 DM was defined as the presence of ICD-10 code E11–E14 and prescription of antidiabetic medications, or a fasting blood glucose (FBG) concentration ≥ 126 mg/dL measured in the NHIS health examination [11]. People having TB before the index year (n = 121, 14) were excluded. Analysis was performed after excluding people with incident TB or death occurring during the first year of follow-up (n = 37,670) to account for the possibility of reverse causation. A total of 96, 936 people with missing data for at least one variable were excluded. Finally, the study population was 2,489,718 individuals with type 2 DM (Additional file 1: Figure S1).

This study was approved by the Institutional Review Board of The Catholic University of Korea (No. SC22ZISE0045). Anonymized and deidentified information was used for analyses and, therefore, informed consent was not obtained.

### Diabetes severity score

The parameters used to produce the diabetes severity score in this study were as follows: complexity of diabetes medications (use of insulin or multiple OHAs); longer duration of diabetes; diabetes-related renal complications (chronic kidney disease, CKD); or the presence of cardiovascular disease (CVD). Each characteristic was treated as one point in the diabetes severity score, and the sum of these (total 0–5) was defined as the diabetes severity score. If ≥ 3 OHAs were used, a diabetes severity score of 1 was recorded, and if the diabetes duration was ≥ 5 years, a diabetes severity score of 1 was recorded. CKD was defined as an estimated glomerular filtration rate (eGFR) < 60 mL/min/1.73 m^2^ [12]. CVD was defined as prior myocardial infarction (I21, I22) or prior stroke (I63, I64) diagnosed as ICD-10 codes in one or more inpatient or outpatient records within 3 years before the index date [13, 14]. For example, a person who used insulin concurrently with CKD had a diabetes severity score of 2. To determine the effect of blood glucose level on incident TB, we also analyzed the risk of TB according to the decile of FBG concentration.

### Measurements and definitions

Covariates were based on the data from the index year and included age, sex, socioeconomic status (income level), body mass index (BMI; kg/m^2^), current smoking status, alcohol consumption, exercise (no/yes), and systolic and diastolic blood pressure (mmHg). Blood samples for the measurement of serum glucose, creatinine, and lipid levels were drawn after an overnight fast. Income level was dichotomized at the lower 25th percentile. The glucose-lowering agents analyzed in this study were grouped into six classes: insulin, sulfonylureas, metformin, thiazolidinediones, dipeptidyl-peptidase IV inhibitors (DPPIV inhibitors), and alpha-glucosidase inhibitors.

### Study outcome: incident TB

The primary study outcome was a new active TB diagnosis, which was defined using the rare intractable disease (RID) registration codes for TB (V206, V246, and V000). The South Korean government has operated a RID registration program (V-code) in serious and costly long-term diseases such as TB to control the disease effectively [13, 14]. Therefore, patients who had the TB-specific V-codes under RID program registration were offered up to 90–100% copayment reduction. In Korea, it is designated by law to report all new TB cases to nearby public health centers with a diagnosis based on ICD-10 code (A15-A19, U88.0-U88.1) by attending physicians. After then, a diagnosis of TB case is reviewed strictly by NHIS and provided the V-codes for the additional insurance coverage to the patients [15, 16]. Before July 2016, V206 and V246 were used for multidrug-resistant TB/extensively drug-resistant TB and drug-susceptible TB and after that, the code of V000 was unified to all type of TB. Under the mandatory reporting system of TB cases and a strict supervision by NHIS, the number of diagnosis codes and incidences of actual TB are almost identical.

The current study collected both pulmonary and extrapulmonary types of active TB. The diseases were diagnosed mainly by microbiological tests for *Mycobacterium tuberculosis* (M. TB) such as TB-PCR or M. TB culture or by clinical evidence based on radiological findings, biochemical results, and histological reports compatible with active TB, in line with Korean TB guidelines [17]. The study population was followed from the baseline to the date of TB diagnosis, censoring (date of death), or until December 31, 2018, whichever came first.

### Statistical analysis

The baseline characteristics are presented as the mean ± standard deviation (SD) or n (%). Participants were classified into six groups according to their diabetes severity score (0–5). The incidence rate of active TB was calculated by dividing the number of incident cases by the total follow-up duration (person-years). The Cox proportional hazards model was used to estimate hazard ratio (HR) and 95% confidence interval (CI) values for the study outcomes according to the diabetes severity score. The proportional hazards assumption was assessed using the Schoenfeld residuals test, with a logarithm of the cumulative hazard functions based on Kaplan–Meier estimates. Over time, there was no significant departure from proportionality in the hazards.

A multivariable-adjusted proportional hazards model was applied: model 1 was unadjusted; model 2 was adjusted for age and sex; model 3 was adjusted further for BMI, income status, alcohol consumption, smoking, regular exercise, FBG concentration, hypertension, and dyslipidemia. The potential effect modification by age, sex, and obesity was evaluated by stratified analysis and interaction testing using the likelihood-ratio test. We performed additional analysis according to the decile of FBG concentration to estimate the effect of glucose level itself on the risk of active TB. Statistical analyses were performed using SAS version 9.4 (SAS Institute Inc., Cary, NC, USA), and a P value < 0.05 was considered to be significant.

## Results

### Baseline characteristics according to the presence of active TB

There were 21,231 cases of active TB during a median follow-up of 6.8 ± 1.59 years. At the baseline, the group of people with incident TB were older (64 vs. 57 years), had a lower prevalence of obesity (BMI; 23.6 vs. 24.9 kg/m^2^), included more men (66.5 vs. 60%), had a higher prevalence of CKD or CVD, and were more likely to be taking insulin treatment or multiple OHAs (Table 1). The proportion of subjects aged ≥ 65 years was higher in patients with TB than those without TB (Table 1).

**Table 1:** Baseline characteristics of the study participants

	Active tuberculosis		
	No	Yes	
N	2,468,487	21,231	
Age (years)	57 (48–66)	64 (54–72)	< 0.001
< 40 years	196,728 (8.0)	782 (3.7)	
40–64 years	1,555,698 (63.0)	10,313 (48.6)	
≥ 65 years	716,061 (29.0)	10,136 (47.7)	
Sex (male)	1,481,340 (60.0)	14,108 (66.5)	< 0.001
Body mass index (kg/m^2^)	24.9 (22.9–27.1)	23.6 (21.6–27.1)	< 0.001
Smoking			< 0.001
Non-smoker	1,368,075 (55.4)	11,044 (52.0)	
Ex-smoker	451,376 (18.3)	3767 (17.7)	
Current smoker	649,036 (26.3)	6420 (30.2)	
Alcohol drinking	251,173 (10.2)	2589 (12.2)	< 0.001
Regular exercise	505,358 (20.5)	4062 (19.1)	< 0.001
Income (lower 25%)	518,074 (21.0)	4825 (22.7)	< 0.001
Dyslipidemia	1,039,708 (42.1)	8121 (38.3)	< 0.001
Hypertension	1,393,988 (56.5)	12,777 (60.2)	< 0.001
Chronic Kidney disease	277,637 (11.3)	3527 (16.6)	< 0.001
Cardiovascular disease	324,264 (13.1)	3698 (17.4)	< 0.001
Duration of diabetes ≥ 5 years	739,450 (30.0)	8623 (40.6)	< 0.001
Pharmacologic therapy for DM			
Insulin	180,411 (2.1)	1274 (11.3)	< 0.001
Number of OHAs ≥ 3	348,593 (14.1)	4205 (19.8)	< 0.001
Medication			
Metformin	1,162,405 (47.1)	11,229 (52.9)	< 0.001
Sulfonylurea	1,001,747 (40.6)	10,966 (51.7)	< 0.001
DPPIV-inhibitors	214,362 (8.7)	1866 (8.8)	0.5882
Thiazolidinedione	155,753 (6.31)	1644 (7.7)	< 0.001
Alpha-glucosidase inhibitors	269,829 (10.9)	3586 (16.9)	< 0.001
Systolic BP (mmHg)	129.2 ± 15.9	129.5 ± 16.5	< 0.001
Diastolic BP (mmHg)	79.2 ± 10.3	78.5 ± 10.4	< 0.001
Fasting glucose (mg/dL)	145.2 ± 47.0	150.9 ± 57.9	< 0.001
eGFR (ml/min/1.73 m^2^)	85.1 ± 36.4	82.4 ± 38.8	< 0.001
Baseline TC (mg/dL)	197.8 ± 46.9	192.0 ± 48.1	< 0.001

Data are expressed as the means ± standard deviation, median (25–75%), or number (percentage, %)

BP, blood pressure; eGFR, estimated glomerular filtration rate; TC, total cholesterol; OHAs, oral hypoglycemic agents; DPPIV-inhibitors, Dipeptidyl-peptidase IV inhibitors

### Risk of active TB according to components of the diabetes severity score

Each parameter of the diabetes severity score was associated with an increased risk of active TB (all P < 0.001). The risk of TB incidence was higher in the group with a diabetes duration of ≥ 5 years (adjusted HR = 1.25; 95% CI, 1.21–1.28) than in that with a diabetes duration < 5 years (Table 2). The risk of TB was 1.28 times higher in participants receiving ≥ 3 OHAs compared with < 3 OHAs. The risk of TB was 1.47 times higher in those treated with insulin compared with those treated without insulin (adjusted HR = 1.47; 95% CI, 1.42–1.54). The overall incidence rates of TB were 1.16 and 1.89 per 1, 000 person-years in participants without CKD and in those with CKD, respectively. Even after controlling for several confounding factors, the presence of CKD was significantly associated with TB risk (adjusted HR = 1.30; 95% CI, 1.25–1.34). The risk of TB increased significantly in participants with CVD compared with those without CVD (adjusted HR = 1.14; 95% CI, 1.10–1.19).

**Table 2:** Risk of active tuberculosis according to the parameters in the diabetes severity score

		Events (n)	Incidence rate (per 1000 person-years)	Model 1*	Model 2*	Model 3*
Duration of diabetes	< 5 years	12, 608	1.05	1 (Ref.)	1 (Ref.)	1 (Ref.)
	≥ 5 years	8623	1.68	1.59 (1.55, 1.63)	1.28 (1.25, 1.32)	1.25 (1.21, 1.28)
Number of Oral hypoglycemic agents	< 3	17, 026	1.16	1 (Ref.)	1 (Ref.)	1 (Ref.)
	≥ 3	4205	1.71	1.47 (1.43, 1.53)	1.34 (1.29, 1.38)	1.28 (1.23, 1.32)
Use of insulin	No	18, 516	1.17	1 (Ref.)	1 (Ref.)	1 (Ref.)
	Yes	2715	2.03	1.73 (1.66, 1.80)	1.56 (1.50, 1.63)	1.47 (1.42, 1.54)
Chronic kidney disease	No	17, 704	1.16	1 (Ref.)	1 (Ref.)	1 (Ref.)
	Yes	3527	1.89	1.63 (1.57, 1.69)	1.24 (1.19, 1.28)	1.30 (1.25, 1.34)
Cardiovascular disease	No	17, 533	1.17	1 (Ref.)	1 (Ref.)	1 (Ref.)
	Yes	3698	1.72	1.47 (1.42, 1.53)	1.08 (1.04, 1.12)	1.14 (1.10, 1.19)

^*^Values are presented as hazard ratio (95% confidence intervals) for risk of tuberculosis

“Ref.” refers to the group treated as a reference group in the analysis

Model 1: unadjusted

Model 2: adjusted for age and sex

Model 3: adjusted for age, sex, BMI, income status, alcohol drinking, smoking, regular exercise, fasting glucose, hypertension, and dyslipidemia

### Incidence and risk of active TB according to the diabetes severity score

**Fig. 1:**
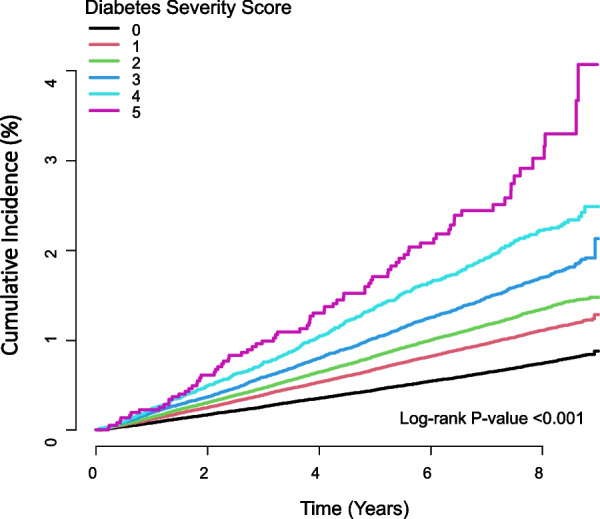
Kaplan–Meier estimates of the cumulative incidence of active tuberculosis (TB) according to the diabetes severity score

Each of the abovementioned characteristics was given one point in the diabetes severity scale, and their sum (0–5) was defined as the diabetes severity score. The risk of TB increased progressively with increasing diabetes severity score (Table 3, Fig. 1). After adjusting for possible confounding factors, the HRs (95% CI) for TB were 1.23 (1.19–1.27) in participants with one parameter, 1.39 (1.33–1.44) in those with two parameters, 1.65 (1.56–1.73) in those with three parameters, 2.05 (1.88–2.23) in those with four parameters, and 2.62 (2.10–3.27) in those with five parameters compared with participants with no parameters.

**Table 3:** Risk of active tuberculosis according to the diabetes severity score

	Total	Events (n)	Incidence rate (per 1000 person-years)	Model 1*	Model 2*	Model 3*
0	1,320,897	8423	0.92	1 (Ref.)	1 (Ref.)	1 (Ref.)
1	636,816	6125	1.39	1.50 (1.45, 1.55)	1.20 (1.16, 1.25)	1.23 (1.19, 1.27)
2	359,679	4143	1.68	1.82 (1.75, 1.89)	1.38 (1.33, 1.44)	1.39 (1.33, 1.44)
3	134,811	1883	2.12	2.30 (2.19, 2.42)	1.64 (1.56, 1.73)	1.65 (1.56, 1.73)
4	33,841	577	2.76	3.00 (2.76, 3.26)	2.04 (1.87, 2.22)	2.05 (1.88, 2.23)
5	3674	80	3.77	4.10 (3.29, 5.11)	2.69 (2.16, 3.36)	2.62 (2.10, 3.27)
*P* for trend				< 0.001	< 0.001	< 0.001

^*^Values are presented as hazard ratio (95% confidence intervals) for risk of tuberculosis

“Ref.” refers to the group treated as a reference group in the analysis

Model 1: unadjusted

Model 2: adjusted for age and sex

Model 3: adjusted for age, sex, BMI, income status, alcohol drinking, smoking, regular exercise, fasting glucose, hypertension, and dyslipidemia

Among the participants with two parameters (score of 2), the highest HR for TB was in those using insulin and multiple OHAs, or in those using insulin and with CKD (Additional file 1: Table S1). Among the participants with three parameters (score of 3), the highest HR for TB was in those who used insulin and had CKD and DM duration of ≥ 5 years (Additional file 1: Table S1).

**Fig. 2:**
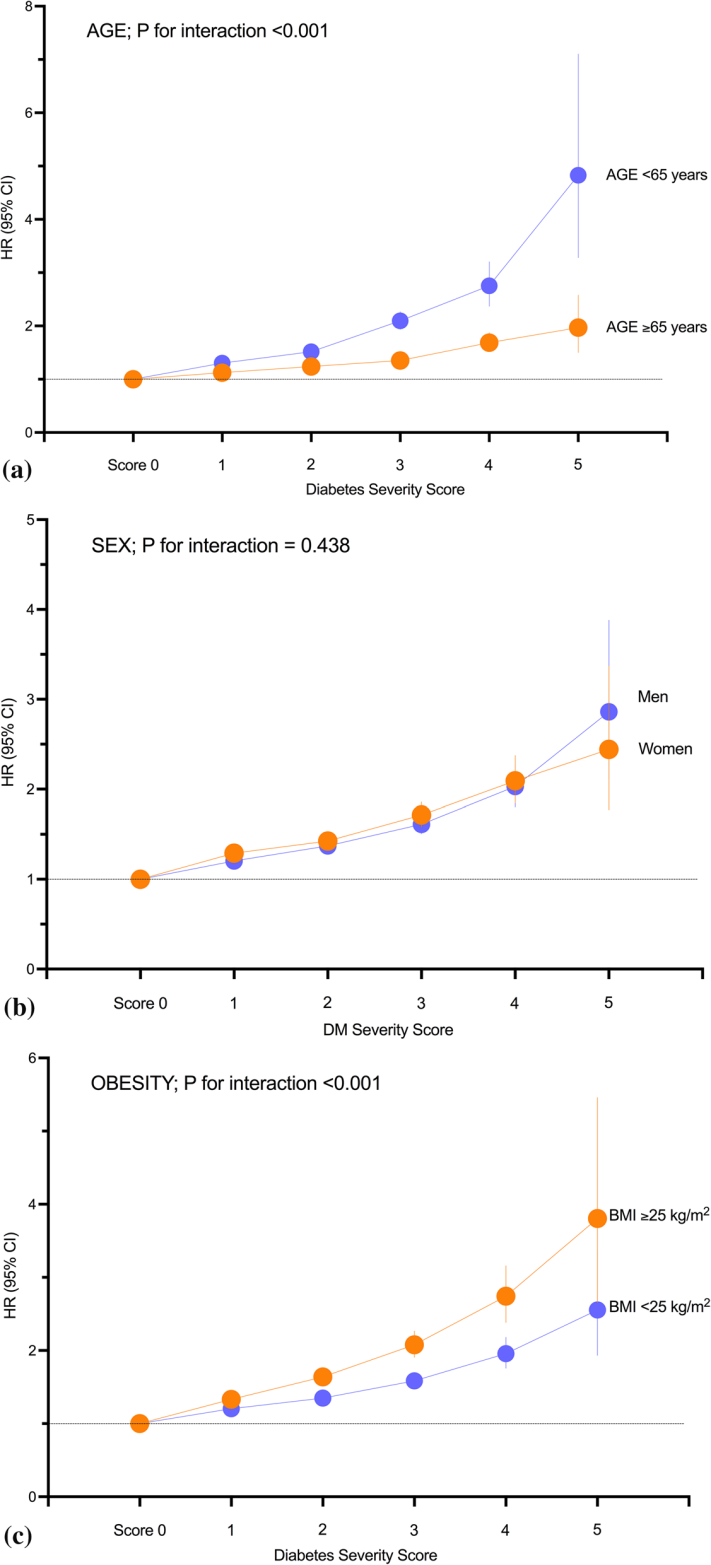
Subgroup analyses of the association between the diabetes severity score and risk of active tuberculosis (TB) stratified by age (**a**), sex (**b**), and obesity (**c**) category. Hazard ratios (HRs) and 95% confidence intervals (CIs) for active TB according to the diabetes severity score. The data were adjusted for age, sex, body mass index, income status, alcohol consumption, smoking, regular exercise, fasting glucose concentration, hypertension, and dyslipidemia

We performed stratified analyses by age, sex, and the presence of obesity. In all subgroups, the risk of TB increased significantly in participants with a higher diabetes severity score compared with those with a score of zero. The effect of a higher diabetes severity score on the risk of TB was stronger in younger participants and in those with obesity (*P* for interaction < 0.001, Fig. 2a, c). The association between diabetes severity scores and TB risk was similar in men and women (*P* for interaction = 0.438, Fig. 2b).

### Effect of fasting blood glucose concentration on the risk of active TB in type 2 diabetes

**Fig. 3:**
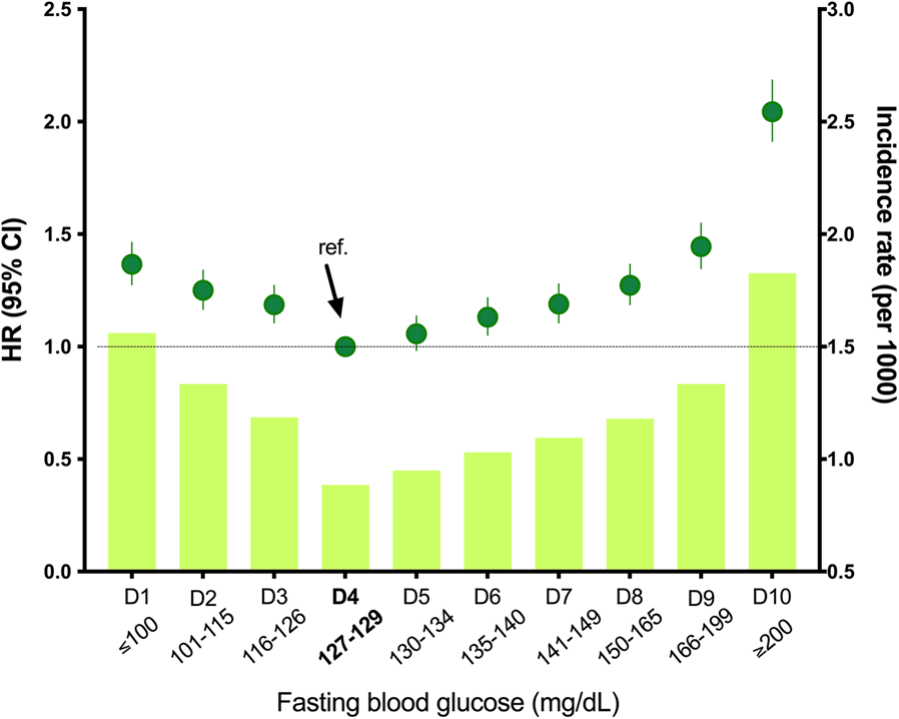
Hazard ratios (HRs) and 95% confidence intervals (CIs) for active tuberculosis (TB) by decile (D) of fasting blood glucose (FBG) concentration. The group with FBG concentration of 127–129 mg/dL (D4) was the reference group. HRs were adjusted for age, sex, body mass index, income status, alcohol consumption, smoking, regular exercise, fasting glucose concentration, hypertension, and dyslipidemia

The lowest risk of TB was observed in the FBG range around 130 mg/dL (D4 and D5). The multivariable adjusted HRs (95% CI) for TB that were associated with FBG levels < 100 (D1), 101–115 (D2), 116–126 (D3), 130–134 (D5), 135–140 (D6), 141–149 (D7), 150–165 (D8), 166–199 (D9), and ≥ 200 (D10) mg/dL were 1.37 (1.28–1.46), 1.25 (1.17–1.34), 1.19 (1.11–1.27), 1.06 (0.98–1.14), 1.13 (1.05–1.22), 1.19 (1.11–1.28), 1.27 (1.19–1.37), 1.45 (1.35–1.55), and 2.04 (1.91–2.19), respectively, compared with 127–129 mg/dL (D4). A J-shaped association was noted between glycemic control and the risk of TB; that is, both low (≤ 115 mg/dL) and high (≥ 150 mg/dL) FBG concentrations conferred a greater than 20% higher risk of TB compared to FBG 127–129 mg/dL (Fig. 3). An FBG level ≥ 200 mg/dL was associated with a twofold increased risk of active TB (Fig. 3).

## Discussion

Since 2011, the World Health Organization (WHO) and the International Union Against Tuberculosis and Lung Disease have highlighted TB–DM co-epidemic and have recommended bidirectional screening [18]. However, it seems difficult to apply such policies fully in the field considering the large population of people with DM worldwide and that most people with both diseases have a low socioeconomic status. In this context, our findings provide evidence for the feasibility of focusing on TB screening in people with DM and a high diabetes severity score.

Previous studies have investigated the impact of indicators of DM severity or status on the development of TB infection. A prospective study of the Taiwanese population found that people with ≥ 2 diabetes-related complications had a threefold increased risk of incident TB [19]. However, the control group was nondiabetic people and the percentage of those with DM was very small, at only 7–9% of all those included in that study [19]. DM is a chronic heterogeneous metabolic disorder that varies in severity from mild to severe. Assessment of DM severity may be helpful for identifying people requiring targeted and intensive therapies, which may provide a major benchmark for efficient healthcare services [6]. In the current study, we applied five parameters to represent diabetes severity, such as use of insulin and/or multiple OHAs, duration of DM ≥ 5 years, and the presence of CVD or CKD, and we found a significant association between the number of parameters and risk of TB. These parameters are easily obtained in real clinical practice, especially in resources-limited settings, because there is no need for laboratory testing.

The FBG concentration is a fundamental element for achieving good glycemic control and managing DM. Previous studies have shown a J-shaped distribution between all-cause mortality and glycemic control in people with DM and that both high and low FBG levels are associated with a higher risk of all-cause mortality [10]. Similarly, the association between FBG concentration and risk of TB was also found to be J-shaped in our study. A previous study also observed that people with poor glycemic control (FBG > 130 mg/dL) had a higher adjusted HR (2.21) for the risk of TB than those without diabetes but that the risk of TB did not differ significantly between diabetic patients with good glycemic control (≤ 130 mg/dL) and nondiabetic people [20]. In our study, we found that the lowest risk of TB was observed in the FBG range around 130 mg/dL. DM-induced hyperglycemia has been suggested to increase the susceptibility to TB infection [16]. However, we also found that an FBG level ≤ 100 mg/dL was associated with a 37% higher risk for active TB. The increased risk for active TB in people with diabetes and an FBG level ≤ 100 mg/dL is consistent with the U-shaped curve between high mortality rates and hypoglycemia in patients in an intensive care unit [21]. The exact mechanism underlying the association between low FBG level and high risk of active TB among patients with type 2 DM remains unclear. One possibility is that low FBG concentration may be a marker of poor nutritional status, liver dysfunction, or other undetectable illnesses [22].

Kidney failure requiring dialysis is recognized as an important risk factor for active TB [23]. Accordingly, the WHO recommends systematic testing and treatment for latent TB infection in this population, a strategy that appears to be safe and effective for reducing the rate of active TB in dialysis patients [24]. Despite a well-established link between dialysis and TB risk, the relationship between CKD before kidney replacement therapy and TB risk requires closer examination. The risk of TB was higher in patients with more severe CKD among patients with CKD stage 2 or higher [25]. Poor immune function in individuals with more profound impairment of kidney function may explain this outcome [25].

TB and atherosclerotic CVD have a close epidemiological and pathogenetic overlap [26]. DM is strongly associated with CVD, and TB infection may increase the risk of CVD further. In one study, TB was associated with an increased risk of acute myocardial infarction (adjusted HR, 1.98; 95% CI, 1.3–3.0) [26]. Another study found that patients with atherosclerotic CVD had higher HR for all-cause and infection-related mortality after initiation of TB treatment [27]. Elevated serum markers of inflammation were found to mediate one-quarter to one-third of this association [27]. There is little evidence that patients with CVD may have an increased risk of active TB. We found that diabetic patients with CVD had a 14% higher risk of active TB compared with those without CVD.

This study has some limitations. First, we may have missed other indicators of DM severity such as diabetic neuropathy or HbA1c level. The clinical manifestations of more severe type 2 DM reflect diverse and complex pathophysiological processes that affect different organ systems over time and make it difficult for a single measure of severity to capture this complexity adequately [6]. We characterized the clinical features indicative of diabetic severity within the range of information we could access. All five parameters in the diabetes severity score in our study showed a significant association with the development of active TB. Second, this was an observational study and, therefore, the association between diabetes severity and active TB may not be causal. To minimize the possible effects of reverse causality, we excluded people with incident TB during the first year of follow-up. Third, this study was restricted to include only the Korean population, where the incidence of TB is 49 cases per 100, 000 people in 2020, relatively high among high-income countries [5], and characteristics associated with DM may differ from those of people of other ethnicities. Fourth, participants in this study were more men than women (60% vs. 40%). Selection of study subjects who participated in health examinations might be a source of bias because men and employee subscribers were more likely to participate in the regular health check-up. Fifth, we were unable to adjust for some unmeasured risk factors, such as contact with a known TB case, which may have mediated the association between DM and TB. Lastly, we did not further analyze the characteristics of TB patients such as a percentage of microbiological diagnosis or severity of TB. According to the National Tuberculosis report of South Korea, pulmonary TB were 79% and extrapulmonary TB were 21% out of the newly diagnosed TB cases. In pulmonary TB, 27.7% cases had positive result for acid-fast stain [28]. This study could not analyze the detailed clinical courses or mortality of subjects with TB. According to a recent study on mortality among TB survivors in Korea, the risk of all-cause mortality was reported to be 1.62 times higher in TB survivors compared to the age- and sex- matched control group [16]. Further studies on the mortality and clinical course of TB in diabetic patients are needed.

In Global TB report 2022, an estimated 0.37 million incident TB cases were attributable to diabetes in 2021 whereas 2.2 million to undernourishment, 0.86 million to HIV infection [29]. As five health-related risk factors for TB such as undernourishment, HIV infection, alcohol use, smoking and diabetes monitored by the WHO have variable impact to TB incidence among the countries or by period, therefore a decision-making and related efforts at a national level are needed that which factors would be prioritized to reduce the TB burden efficiently.

To our knowledge, this is the largest diabetes cohort study over a 6-year follow-up. We ascertained that the diabetes severity score, which included measures of the use of insulin and multiple OHAs, duration of diabetes, and the presence of CKD or CVD, was associated with TB risk in a dose-dependent manner. Of the components included in the diabetes severity score, insulin use was the most significant factor related to the risk of TB, followed by CKD. In addition, in people with type 2 DM, the FBG concentration had a J-shaped relationship with TB occurrence, and the lowest risk of TB was observed in those with an FBG concentration around 130 mg/dL. Both hyperglycemia and hypoglycemia were associated with a higher risk of TB development. These findings suggest that people with a higher diabetes severity score, particularly insulin users or patients with CKD, may be a targeted group for additional active TB screening in clinical practice.

## Supplementary Information

**Additional file 1: Table S1.** Incidence rates and hazard ratios (HRs) of active tuberculosis (TB) according to the diabetes severity score and components of the diabetes severity score. **Figure S1.** Flow chart of the Study population.

## Data Availability

The datasets used and/or analyzed in the current study are available from the corresponding author upon reasonable request.
