# Supplementary material for: Diabetes severity is strongly associated with the risk of active tuberculosis in people with type 2 diabetes: a nationwide cohort study with a 6-year follow-up
Source: Respir Res. 2023 Apr 11;24:110. doi: 10.1186/s12931-023-02414-5 (PMC10088122; doi:10.1186/s12931-023-02414-5)
Supplement: Supplementary file 1 — Additional file 1: Table S1. Incidence rates and hazard ratios (HRs) of active tuberculosis (TB) according to the diabetes severity score and components of the diabetes severity score. Figure S1. Flow chart of the Study population. [file 12931_2023_2414_MOESM1_ESM.docx]

**Table S1.** Incidence rates and hazard ratios (HRs) of active tuberculosis (TB) according to the diabetes severity score and components of the diabetes severity score.

| Diabetes Severity Score | DM duration ≥ 5 years | Number of OHAs ≥ 3 | Use of Insulin | Chronic kidney disease | Cardiovascular  disease | Incidence rate (per 1000 person-years) | HR (95% CI) |
| --- | --- | --- | --- | --- | --- | --- | --- |
| 0 |  |  |  |  |  | 0.92 | 1 (ref) |
| 1 |  |  |  |  | Y | 1.35 | 1.15(1.08,1.23) |
| 1 | Y |  |  |  |  | 1.40 | 1.20(1.15,1.25) |
| 1 |  |  |  | Y |  | 1.41 | 1.24(1.16,1.33) |
| 1 |  | Y |  |  |  | 1.26 | 1.32(1.22,1.42) |
| 1 |  |  | Y |  |  | 1.57 | 1.50(1.35,1.65) |
| 2 | Y |  |  |  | Y | 1.59 | 1.22(1.13,1.32) |
| 2 |  | Y |  | Y |  | 1.45 | 1.26(1.01,1.57) |
| 2 | Y | Y |  |  |  | 1.61 | 1.37(1.30,1.45) |
| 2 |  | Y |  |  | Y | 1.54 | 1.37(1.14,1.66) |
| 2 | Y |  |  | Y |  | 1.83 | 1.41(1.29,1.54) |
| 2 |  |  |  | Y | Y | 1.95 | 1.43(1.27,1.60) |
| 2 | Y |  | Y |  |  | 1.64 | 1.44(1.32,1.57) |
| 2 |  |  | Y |  | Y | 1.77 | 1.44(1.17,1.78) |
| 2 |  |  | Y | Y |  | 2.23 | 1.85(1.48,2.31) |
| 2 |  | Y | Y |  |  | 2.18 | 1.96(1.68,2.28) |
| 3 | Y | Y |  |  | Y | 1.79 | 1.37(1.23,1.53) |
| 3 |  | Y | Y | Y |  | 1.99 | 1.49(0.97,2.29) |
| 3 | Y |  | Y |  | Y | 1.90 | 1.50(1.31,1.73) |
| 3 |  | Y |  | Y | Y | 2.06 | 1.61(1.11,2.34) |
| 3 | Y |  |  | Y | Y | 2.32 | 1.64(1.45,1.85) |
| 3 | Y | Y |  | Y |  | 2.15 | 1.65(1.47,1.84) |
| 3 | Y | Y | Y |  |  | 2.08 | 1.66(1.49,1.85) |
| 3 |  | Y | Y |  | Y | 2.09 | 1.73(1.21,2.48) |
| 3 |  |  | Y | Y | Y | 2.72 | 1.99(1.43,2.76) |
| 3 | Y |  | Y | Y |  | 2.78 | 2.31(2.05,2.61) |
| 4 | Y | Y |  | Y | Y | 2.35 | 1.67(1.41,1.99) |
| 4 |  | Y | Y | Y | Y | 2.81 | 1.93(1.04,3.60) |
| 4 | Y | Y | Y |  | Y | 2.82 | 2.04(1.73,2.40) |
| 4 | Y | Y | Y | Y |  | 3.03 | 2.25(1.87,2.70) |
| 4 | Y |  | Y | Y | Y | 2.95 | 2.31(1.98,2.69) |
| 5 | Y | Y | Y | Y | Y | 3.77 | 2.62(2.10,3.27) |

Adjusted for age, sex, BMI, income status, alcohol drinking, smoking, regular exercise, fasting glucose, hypertension, and dyslipidemia

CI, confidence intervals; DM, diabetes mellitus; HR, hazard ratio; OHAs, oral hypoglycemic agents.

**Figure S1.** Flow chart of the Study population

**Followed from index year to the date of TB diagnosis, death or until Dec 31, 2018**

Median Follow-up: 6.8 years

37670 subjects having incident TB or death within the first year after the index (1-year lag period) were excluded.

n = 2,489,718

Subjects eligible for inclusion

121,314 subjects having prior TB before the index year were excluded.

96,936 subjects were excluded with missing data on at least one variable.

2,745,638

Participants (≥ 20yrs) who had undergone a health examination during 2009-2012
